# Impact of various types of near work and time spent outdoors at different times of day on visual acuity and refractive error among Chinese school-going children

**DOI:** 10.1371/journal.pone.0215827

**Published:** 2019-04-26

**Authors:** Hongyu Guan, Ning Neil Yu, Huan Wang, Matthew Boswell, Yaojiang Shi, Scott Rozelle, Nathan Congdon

**Affiliations:** 1 Center for Experimental Economics for Education, Shaanxi Normal University, Xi’an, China; 2 Freeman Spogli Institute of International Studies, Stanford University, Stanford, CA, United States of America; 3 Centre for Public Health, Queen's University Belfast, Belfast, United Kingdom; 4 Orbis International, New York, NY, United States of America; 5 Zhongshan Ophthalmic Center, Guangzhou, China; Sun Yat-Sen University Zhongshan Ophthalmic Center, CHINA

## Abstract

**Background:**

Various types of near work have been suggested to promote the incidence and progression of myopia, while outdoor activity appears to prevent or retard myopia. However, there is a lack of consensus on how to interpret these results and translate them into effective intervention strategies. This study examined the association between visual acuity and time allocated to various activities among school-going children.

**Methods:**

Population-based survey of 19,934 students in grade 4 and 5 from 252 randomly selected rural primary schools in Northwest China in September 2012. This survey measured visual acuity and collected self-reported data on time spent outdoors and time spent doing various types of near activities.

**Results:**

Prolonged (>60 minutes/day) computer usage (-0.025 LogMAR units, P = .011) and smartphone usage (-0.041 LogMAR units, P = .001) were significantly associated with greater refractive error, while television viewing and after-school study were not. For time spent outdoors, only time around midday was significantly associated with better uncorrected visual acuity. Compared to children who reported no midday time outdoors, those who spent time outdoors at midday for 31–60 minutes or more than 60 minutes had better uncorrected visual acuity by 0.016 LogMAR units (P = .014) and 0.016 units (P = .042), respectively.

**Conclusions:**

Use of smart phones and computers were associated with declines in children’s vision, while television viewing was not. Statistically significant associations between outdoor time at midday and reduced myopia may support the hypothesis that light intensity plays a role in the protective effects of outdoor time.

## Introduction

Various types of near work[1–4] have been suggested to promote the incidence and progression of myopia[2,5–8], while outdoor activity appears to prevent or retard myopia[9]. However, there is a lack of consensus on how to interpret these results and to translate them into effective intervention strategies[10–14].

One difficulty lies in differentiating the direct effects of more time spent on one activity, as well as the substitution effects of having less time left for other activities[10]. Spending more time outdoors may reduce myopia because children spend less time on near work, while spending more time on near work may exacerbate myopia. Attempts have been made to account for this difficulty in a few studies, but it remains relevant to interpreting the body of literature on the impact of time allocation. Another difficulty lies in elucidating the effect on myopia of different factors underlying an activity. For example, time spent outdoors may lead both to increased light exposure and a tendency to focus on more distant objects, each of which might potentially reduce myopia risk.

The current study reports data from a large population-based survey of rural primary school students in Northwest China, during which visual acuity was measured and a widely-used form[15] was utilized to collect self-reported data on time spent outdoors and in various types of near activities.

We used a diary approach to collect self-reported data on time spent outdoors; this not only allowed us to identify potential substitution effects, but also to distinguish between time spent outdoors before school, around midday, and after school in order to test the hypothesis that brighter light, rather than a tendency to focus at greater distance, is responsible for the protective effect of outdoor activities against myopia[16]. This is because the level of illumination from the sun is strongest around midday, and weaker in the morning and late afternoon hours before or after school, while the tendency to focus at greater distance is presumably unchanged throughout the day.

This paper also examines the association between myopia and uncorrected visual acuity, and between myopia and various types of near activities, including schoolwork and the use of smartphones, computers and televisions, in order to guide potential interventions to reduce the visually-harmful impact of such activities.

## Methods

### Setting and sampling

This study utilizes data collected during a randomized trial of glasses provision on educational outcomes among 19,934 students at 252 primary schools in Northwest China, in the fall of 2012.

Our sample schools are located in A prefecture in G province and B Prefecture in S province in northwest of China. We obtained a list of all 435 primary schools in the two prefectures from local education bureaus. For logistical reasons we excluded those with fewer than 50 or more than 150 students in the fourth and fifth grade combined (19% of sample frame). This is because screening at the larger schools could not be reliably completed in a day, which would have interfered with the screening schedule, whereas smaller schools would be expected to have fewer than 10 children requiring glasses, below our power requirements. We randomly selected one school from each township in the sample, and within each school we randomly selected one class in each of the fourth and fifth grades (likely age range 9–12 years). All 19,934 students in these 252 schools completed a detailed questionnaire concerning potential risk factors for myopia, including weekly time spent in near activities and time spent outdoors.

Institutional Review Boards at Stanford University and Zhongshan Ophthalmic Center (ZOC, Sun Yat-sen University, Guangzhou, China) approved the research protocol in full, and the principles of the Declaration of Helsinki were followed throughout. Written informed consent was obtained from at least one parent for all child participants.

### Assessment of Visual Acuity (VA), refraction, and myopia

In each school, a nurse and staff assistant, who had been trained by optometrists from ZOC, carried out visual acuity (VA) screening for all selected students to determine eligibility for the original trial. In a well-lighted indoor space at each school, children underwent assessment of uncorrected VA (UCVA) in each eye separately at 4 meters, using an Early Treatment Diabetic Retinopathy Study (ETDRS) chart (Precision Vision, La Salle, Illinois, USA)[15]. If a child correctly identified at least 4 of 5 optotypes on the top 6/60 line, s/he was examined on the 6/30 line, the 6/15 line, and then line by line to 6/3. When a student failed a line, the lines above were tested successively until the child correctly identified 4 of 5 optotypes; the VA for an eye was defined as the lowest line read. For cases in which a student could not correctly read the top line, visual acuity was tested as above at 1 meter, with the measured VA divided by 4. VA was expressed during data analysis using LogMAR, the negative base-10 logarithm of the Minimum Angle of Resolution.

If a child’s VA was ≤6/12 in either eye, cycloplegia was applied with up to 3 drops of cyclopentolate 1%, and a refractionist, previously trained by ZOC’s experienced pediatric optometrists, carried out automated refraction (Topcon KR 8900; Tokyo, Japan) with subjective refinement in each eye separately. Refractive error was recorded as the spherical equivalent (SE, spherical power + ½ the cylindrical power). Myopia was defined as VA ≤6/12 and SE ≤-0.5D in at least one eye.

A total of 19934 students underwent vision screening, 4939 (24.8%) of them failed. Among these who failed the vision screening, 280 (5.7%) students did not complete refraction due to absence on the day our optometrists team conducted cycloplegic refraction in school. The remaining 4659 (94.3%) completed cycloplegic refraction. Students that did not complete cycloplegic refraction and those who did were not significantly different with regard to individual characteristics or time allocation variables.

**Fig 1:**
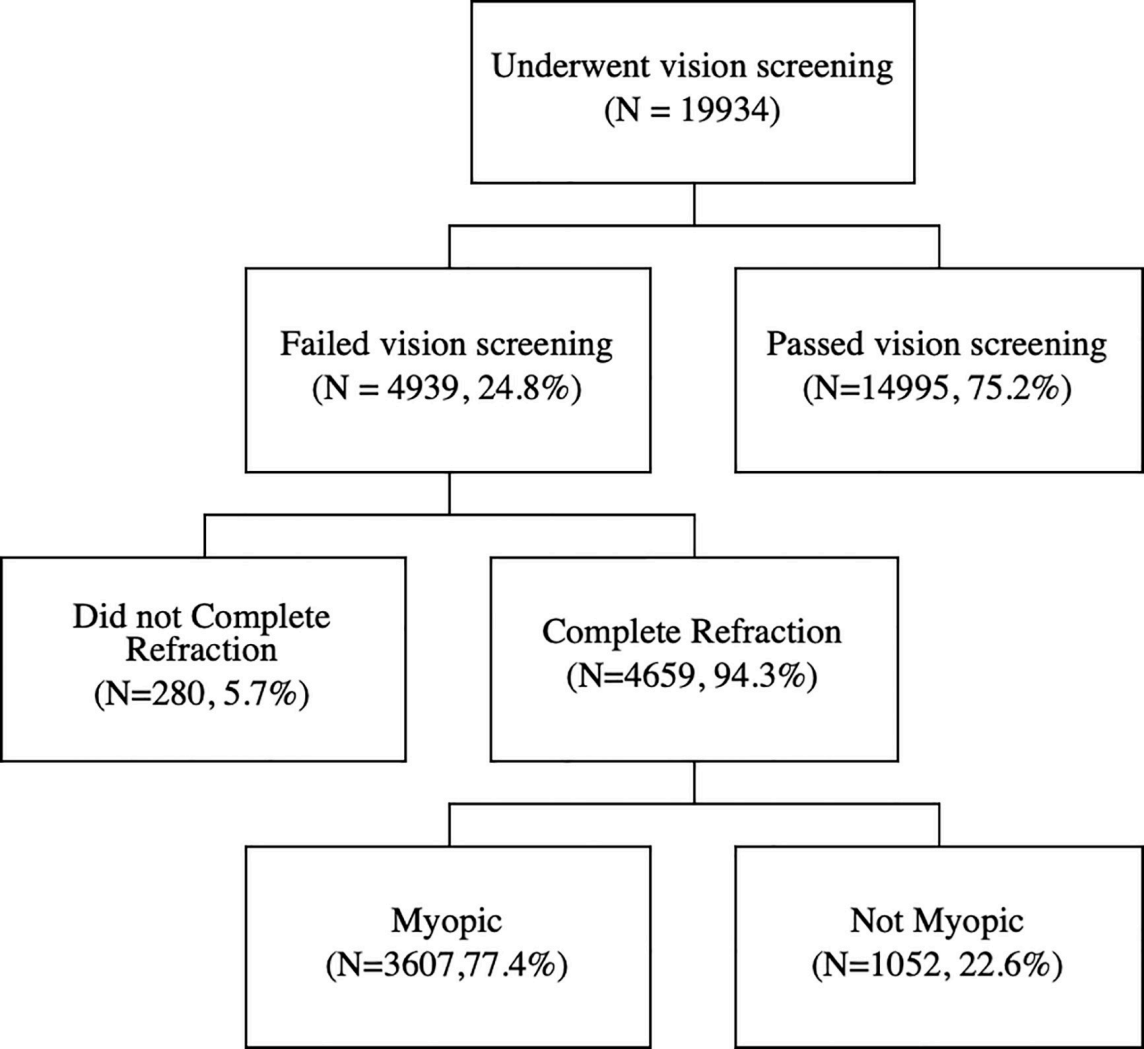
Flowchart of the participants. (A)19934 students underwent vision screening,4939 (24.8%) of them failed, (B) 4659 (94.3%) students that completed cycloplegic refraction, (C) 3607 (77.4%) were diagnosed with myopia.

Among the 4659 (94.3%) students that completed cycloplegic refraction, 1052 (22.6%) of them were not diagnosed with myopia due to visual acuity not being correctable to 6/12 in at least one eye with refraction, due to amblyopia or other non-refractive disorders. These children were referred to higher level hospitals (prefectural and municipal facilities) for treatment. In our analysis, we define these children as not myopic, because their vision could not be improved with refraction. Fig 1 presents a flowchart of the participants in this study.

### Assessment of self-reported time spent in near activities and outdoors

Students completed a previously-reported, self-administered questionnaire concerning mean times spent throughout the day on near activities (including computer and smartphone use, television viewing and after-school study), and time spent outdoors before school, around midday and after school. Reports of time spent on various activities at different periods in the day were categorized as follows: 0 minutes, 1 to 30 minutes, 31 to 60 minutes and > 60 minutes.

### Covariates

Our questionnaire also collected additional information potentially related to myopia, including grade (4^th^ or 5^th^), age, gender, family wealth, migration status of each parent, parental education, child’s main residence (home, school dormitory, relative’s home, etc.), and province. Family wealth was calculated based on a parental questionnaire that asked about ownership of 13 selected items, summing their values as listed in the China Rural Household Survey Yearbook (Department of Rural Surveys, National Bureau of Statistics of China, 2013). S province’s GDP per capita of USD 6108 ranked 14^th^ among China’s administrative regions in 2012, and was very similar to that for the country as a whole (USD 6091) in the same year, while G province was the second-poorest province in the country (per capita GDP of USD 3100)[17]. Higher income is a risk factor for myopia[13], and thus province of residence was explored as a potential determinant of VA and refractive error.

### Statistical methods

Mean UCVA (LogMAR averaged over two eyes, with its 95% Confidence Interval) and myopia prevalence are reported for children stratified by various demographic and behavioral factors. The impact of time allocation and other variables on UCVA in cross-sectional fashion were assessed using Generalized Estimating Equation (GEE) regression models, which adjusted for the correlation between the two eyes of each child and between children in the same school. In the multivariate model (full regression model), to improve the efficiency of the estimation, we included variables which were statistically significant associated with visual acuity and myopia (age, sex, family wealth, parental migrant status, parental education, child’s residence). Missing observations, which constituted a small proportion of the data, were excluded. For statistical interference, robust standard errors adjusted for clustering at the school level were used. Similarly, we constructed logistic models with outcome defined as the presence of myopia and included the same potential explanatory variables. All analyses were performed using Stata 14.0 (StataCorp, College Station, Texas, USA).

## Results

Among the 19,934 children in our study (mean age 10.6 +/-1.15 years, 48% girls), the mean LogMAR UCVA was -0.172, equivalent to a Snellen fraction of approximately 6/9. A total of 4939 children (24.8%) failed vision testing, and among them, 4659 (94.3%) underwent refraction. The prevalence of myopia was 18.1% (3607 of 19,934 students).

Students in grade 5, girls, those from wealthier families, those with at least one parent at home, those with better-educated patents and S province residents had significantly (P < .001) worse UCVA and greater myopia. Greater computer use (P < .001), smartphone use, television viewing, and after-school study as well as less midday outdoor time, were also associated with lower UCVA and greater myopia prevalence (P < .001). Before- and after-school outdoor time were unassociated with UCVA and myopia (Table 1).

**Table 1:** Visual acuity by student characteristics.

CHARACTERISTICS	*n*	*Mean Uncorrected Visual Acuity (LogMAR*^a^*)*	*95% Confidence interval*^b^	*Myopia (VA ≤6/12 and SE ≤-0*.*5D in at least one eye)*	*Missing Data (%)*
All Students	19,934	-0.172	-0.175 - -0.169	18.1%	0(0)
Grade					0(0)
4	9,842	-0.156	-0.160 - -0.152	14.8%	
5	10,092	-0.188	-0.193 - -0.183	21.3%	
Age					18(0.09%)
< = 10	10,304	-0.174	-0.178 - -0.169	18.4%	
>10	9,630	-0.170	-0.175 - -0.165	17.7%	
Sex					
Girls	9,548	-0.189	-0.194 - -0.184	19.6%	0(0)
Boys	10,386	-0.157	-0.161 - -0.152	16.7%	
Family Wealth					924(4.6%)
Bottom tercile	5,573	-0.153	-0.159 - -0.147	14.3%	
Middle tercile	7,024	-0.173	-0.179 - -0.167	17.7%	
Top tercile	6,413	-0.188	-0.194 - -0.182	21.8%	
Parental Migration Status					187(0.9%)
Both out-migrated	2,474	-0.148	-0.157 - -0.139	18.6%	
One or more present	17,273	-0.175	-0.179 - -0.172	14.6%	
Maternal Education					127(0.6%)
Less than junior high school	4,304	-0.162	-0.169 - -0.155	17.0%	
Junior high school	13,784	-0.171	-0.175 - -0.167	18.0%	
At least high school	1,719	-0.205	-0.217 - -0.192	21.3%	
Paternal Education					72(0.4%)
Less than junior high school	1,363	-0.163	-0.175 - -0.151	17.1%	
Junior high school	15,861	-0.170	-0.173 - -0.166	17.8%	
At least high school	2,638	-0.190	-0.200 - -0.181	20.2%	
Child’s main residence					852(4.3%)
Home	11,019	-0.162	-0.167 - -0.158	16.2%	
School dormitory	4,081	-0.182	-0.190 - -0.175	21.9%	
Relative’s home	323	-0.166	-0.190 - -0.142	16.1%	
Rental home	3,641	-0.191	-0.199 - -0.183	19.8%	
Other	18	-0.230	-0.386 - -0.074	16.7%	
Province					0(0)
S province	9,625	-0.206	-0.211 - -0.201	24.3%	
G province	10,309	-0.141	-0.145 - -0.136	12.3%	
Daily Computer Use					0(0)
0 minutes	15,493	-0.160	-0.164 - -0.157	16.5%	
1–30 minutes	2,634	-0.207	-0.217 - -0.297	22.4%	
31–60 minutes	998	-0.217	-0.233 - -0.201	27.2%	
> 60 minutes	809	-0.232	-0.250 - -0.213	23.5%	
Daily Smartphone Use					0(0)
0 minutes	13,161	-0.166	-0.170 - -0.162	17.5%	
1–30 minutes	5,360	-0.180	-0.186 - -0.173	19.4%	
31–60 minutes	829	-0.184	-0.201 - -0.167	18.0%	
> 60 minutes	584	-0.216	-0.237 - -0.194	20%	
Television Viewing Time					0(0)
0 minutes	2,205	-0.170	-0.180 - -0.160	16.4%	
1–30 minutes	8,765	-0.161	-0.166 - -0.156	16.8%	
31–60 minutes	4,765	-0.179	-0.186 - -0.172	19.9%	
> 60 minutes	4,199	-0.189	-0.196 - -0.181	19.8%	
After-School Study Time					0(0)
0 minutes	665	-0.159	-0.176 - -0.141	14.0%	
1–30 minutes	6,227	-0.162	-0.168 - -0.156	16.1%	
31–60 minutes	7,259	-0.169	-0.175 - -0.164	18.0%	
> 60 minutes	5,783	-0.189	-0.195 - -0.182	20.8%	
Before-School Outdoor Time					0(0%)
0 minutes	1,427	-0.171	-0.183 - -0.159	17.2%	
1–30 minutes	15,782	-0.174	-0.178 - -0.170	18.3%	
31–60 minutes	1,710	-0.154	-0.165 - -0.143	17.1%	
> 60 minutes	1,015	-0.173	-0.188 - -0.158	18.0%	
Mid-Day Outdoor Time					855(4.3%)
0 minutes	6,200	-0.184	-0.190 - -0.178	19.5%	
1–30 minutes	9,770	-0.170	-0.174 - -0.165	18.0%	
31–60 minutes	1,958	-0.155	-0.165 - -0.145	15.3%	
> 60 minutes	1,151	-0.157	-0.171 - -0.144	16.0%	
After-School Outdoor Time					860(4.3%)
0 minutes	4,701	-0.173	-0.180 - -0.167	17.3%	
1–30 minutes	9,356	-0.170	-0.175 - -0.166	17.9%	
31–60 minutes	2,915	-0.174	-0.183 - -0.165	19.3%	
> 60 minutes	2,102	-0.175	-0.185 - -0.164	19.3%	

a LogMAR = negative of the base-10 log of the Minimum Angle of Resolution

b Based on LogMAR averaged over two eyes for each individual

In multivariate regression models (Table 2), we found that using a computer 1 to 30 minutes and 31 to 60 minutes per day were associated with worse UCVA (0.019 LogMAR units, P = .001 and 0.024 LogMAR units, P = .008), while greater than 60 minutes of daily use was associated with a greater reduction in UCVA (0.040 LogMAR units, P< 0.001). Using smartphones for 1 to 30 minutes and 31 to 60 minutes per day was uncorrelated with visual acuity, but use for greater than 60 minutes was associated with reduced UCVA (0.043 LogMAR units, P< 0.001). Neither television viewing nor after-school study were significantly associated with declines in UCVA.

**Table 2:** Effects of various factors on visual acuity (LogMAR of both eyes^a^) in Generalized Estimating Equations (GEE)^b^ regression models.

	*Univariate Model*			*Multivariate Model* *(n = 35*,*828)*		
VARIABLES	*beta*	*95% CI*	*p-value*^c^	*beta*	*95% CI*	*p-value*
Grade	-0.030	-0.039 - -0.022	(< 0.001)	-0.033	-0.044 - -0.023	(< 0.001)
Age	-0.003	-0.006–0.000	(0.070)	0.002	-0.002–0.006	(0.356)
Male sex	0.037	0.030–0.043	(< 0.001)	0.038	0.030–0.045	(< 0.001)
Family Wealth	-0.000	-0.000 - -0.000	(0.004)	-0.000	-0.000–0.000	(0.179)
Both Parents Out-Migrated for Work	0.009	-0.001–0.018	(0.066)	0.010	-0.000–0.019	(0.053)
Maternal Education (Less than junior high school as reference)						
Junior high school	-0.002	-0.010–0.007	(0.681)	-0.000	-0.010–0.010	(0.991)
At least high school	-0.023	-0.037 - -0.008	(0.002)	-0.013	-0.029–0.004	(0.131)
Paternal Education (Less than junior high school as reference)						
Junior high school	-0.002	-0.015–0.011	(0.777)	-0.001	-0.014–0.013	(0.939)
At least high school	-0.016	-0.032 - -0.000	(0.050)	-0.004	-0.021–0.013	(0.619)
Child’s Main Residence (Home as reference)						
School dormitory	0.013	0.001–0.024	(0.029)	0.020	0.008–0.032	(0.001)
Relative’s home	-0.004	-0.028–0.019	(0.726)	-0.005	-0.030–0.019	(0.667)
Rental home	0.009	-0.003–0.021	(0.146)	0.010	-0.002–0.023	(0.106)
Other	-0.086	-0.216–0.044	(0.196)	-0.122	-0.273–0.028	(0.110)
G province	0.064	0.047–0.080	(< 0.001)	0.065	0.048–0.083	(< 0.001)
Time Using Computers (0 minutes as reference)						
1–30 minutes	-0.019	-0.030 - -0.008	(0.001)	-0.013	-0.026 - -0.001	(0.041)
31–60 minutes	-0.024	-0.042 - -0.006	(0.008)	-0.019	-0.038–0.001	(0.058)
>60 minutes	-0.040	-0.058 - -0.022	(< 0.001)	-0.025	-0.045 - -0.006	(0.011)
Time Using Smartphones (0 minutes as reference)						
1–30 minutes	-0.004	-0.012–0.003	(0.264)	-0.005	-0.013–0.003	(0.217)
31–60 minutes	-0.007	-0.026–0.012	(0.483)	-0.011	-0.031–0.008	(0.260)
>60 minutes	-0.043	-0.066 - -0.020	(< 0.001)	-0.041	-0.066 - -0.016	(0.001)
Television Viewing Time (0 minutes as reference)						
1–30 minutes	0.010	-0.001–0.021	(0.084)	0.011	-0.001–0.023	(0.080)
31–60 minutes	-0.001	-0.014–0.012	(0.838)	0.003	-0.011–0.017	(0.666)
>60 minutes	-0.007	-0.020–0.006	(0.290)	-0.003	-0.016–0.011	(0.680)
After-School Study Time (0 minutes as reference)						
1–30 minutes	0.004	-0.015–0.023	(0.700)	-0.003	-0.022–0.016	(0.738)
31–60 minutes	-0.000	-0.019–0.019	(0.994)	-0.002	-0.020–0.017	(0.866)
>60 minutes	-0.017	-0.036–0.002	(0.082)	-0.016	-0.036–0.003	(0.099)
Before-School Outdoor Time (0 minutes as reference)						
1–30 minutes	0.077	-0.066–0.220	(0.292)	-0.009	-0.026–0.007	(0.268)
31–60 minutes	-0.007	-0.193–0.180	(0.945)	-0.001	-0.020–0.017	(0.880)
>60 minutes	0.059	-0.152–0.270	(0.581)	-0.013	-0.054–0.028	(0.523)
Mid-Day Outdoor Time (0 minutes as reference)						
1–30 minutes	0.007	-0.001–0.016	(0.079)	0.008	-0.001–0.016	(0.089)
31–60 minutes	0.016	0.003–0.028	(0.012)	0.016	0.003–0.029	(0.014)
>60 minutes	0.019	0.004–0.034	(0.011)	0.016	0.001–0.032	(0.042)
After-School Outdoor Time (0 minutes as reference)						
1–30 minutes	0.006	-0.001–0.014	(0.111)	0.004	-0.004–0.012	(0.361)
31–60 minutes	0.007	-0.004–0.018	(0.195)	0.004	-0.008–0.016	(0.517)
>60 minutes	0.009	-0.003–0.021	(0.136)	0.005	-0.007–0.018	(0.410)

^a^ LogMAR = negative of the base-10 log of the Minimum Angle of Resolution

^b^ GEE adjusts for the correlation between eyes of a child.

^c^ Confidence intervals and *p*-values are based on robust standard errors adjusted for clustering at the school level.

Our logistic regression models with myopia as the outcome (Table 2) showed consistent results for computer use and increased myopia risk, though smartphone usage was not significantly associated with visual acuity. As with the model using UCVA, television viewing and after-school study were unassociated with myopia risk. Such findings are consistent with those found in the literature[18–22].

Regarding time spent outdoors, statistically significant associations between outdoor time and reduced myopia were found only for the midday interval. Myopia prevalence was highest in students who spent the least time outdoors around noon (Table 1), though no such trend was present for before or after school. In the linear regression model for UCVA, children spending 30 to 60 minutes outdoors at noon per day (0.016 LogMAR units, P = .014) and those with greater than 60 minutes outdoors at noon per day (0.016 LogMAR units, P = .042) had significantly better vision compared to children who rarely went outdoors at this time; these differences for other times of day were not statistically significant (Table 2). Results for myopia risk were consistent: only spending more time outdoors at midday had a significant protective effect (Table 3).

**Table 3:** Effects of various factors on myopia (VA ≤ 6/12 and SE < = -0.5D in at least one eye) in logistic regression models.

	*Univariate Model* *(n = 17*,*914)*			*Multivariate Model* *(n = 17*,*914)*		
VARIABLES	*beta*	*95% CI*	*p-value* ^a^	*beta*	*95% CI*	*p-value*
Grade	0.448	0.374–0.521	(< 0.001)	0.509	0.419–0.598	(< 0.001)
Age	-0.031	-0.062 - -0.000	(0.049)	-0.063	-0.105 - -0.020	(0.004)
Male sex	-0.198	-0.270 - -0.126	(< 0.001)	-0.218	-0.298 - -0.137	(< 0.001)
Family Wealth	0.000	0.000–0.000	(< 0.001)	0.000	0.000–0.000	(< 0.001)
Both Parents Out-Migrated for Work	-0.294	-0.413 - -0.176	(< 0.001)	-0.155	-0.283–0.027	(0.017)
Maternal Education (Less than junior high school as reference)						
Junior high school	0.069	-0.021–0.160	(0.681)	0.003	-0.097–0.102	(0.959)
At least high school	0.281	0.141–0.421	(0.002)	0.041	-0.122–0.204	(0.619)
Paternal Education (Less than junior high school as reference)						
Junior high school	0.051	-0.096–0.197	(0.777)	0.033	-0.128–0.193	(0.689)
At least high school	0.205	0.035–0.375	(0.050)	0.089	-0.103–0.282	(0.363)
Child’s Main Residence (Home as reference)						
School dormitory	0.373	0.283–0.463	(0.029)	-0.184	-0.294 - -0.074	(0.001)
Relative’s home	-0.008	-0.309–0.293	(0.726)	-0.030	-0.351–0.291	(0.854)
Rental home	0.244	0.148–0.340	(0.146)	-0.069	-0.176–0.038	(0.208)
Other	0.033	-1.207–1.274	(0.196)	0.335	-1.152–1.823	(0.659)
G province	-0.827	-0.902 - -0.752	(< 0.001)	-0.785	-0.886 - -0.683	(< 0.001)
Time Using Computers (0 minutes as reference)						
1–30 minutes	0.379	0.278–0.480	(< 0.001)	0.017	-0.097–0.131	(0.776)
31–60 minutes	0.635	0.489–0.780	(< 0.001)	0.305	0.141–0.468	(< 0.001)
>60 minutes	0.440	0.272–0.608	(< 0.001)	0.032	-0.161–0.226	(0.744)
Time Using Smartphones (0 minutes as reference)						
1–30 minutes	0.131	0.049–0.212	(0.002)	0.025	-0.065–0.115	(0.591)
31–60 minutes	0.034	-0.149–0.217	(0.714)	-0.015	-0.215–0.185	(0.885)
>60 minutes	0.168	-0.039–0.376	(0.112)	0.161	-0.068–0.391	(0.168)
Television Viewing Time (0 minutes as reference)						
1–30 minutes	0.032	-0.094–0.158	(0.619)	-0.040	-0.177–0.097	(0.565)
31–60 minutes	0.239	0.106–0.373	(< 0.001)	0.031	-0.117–0.178	(0.685)
>60 minutes	0.235	0.099–0.372	(0.001)	0.067	-0.086–0.220	(0.394)
After-School Study Time (0 minutes as reference)						
1–30 minutes	0.170	-0.060–0.400	(0.148)	0.101	-0.148–0.349	(0.428)
31–60 minutes	0.305	0.077–0.534	(0.009)	0.176	-0.071–0.423	(0.163)
>60 minutes	0.483	0.254–0.713	(< 0.001)	0.318	0.070–0.566	(0.012)
Before-School Outdoor Time (0 minutes as reference)						
1–30 minutes	0.077	-0.066–0.220	(0.292)	-0.011	-0.162–0.139	(0.883)
31–60 minutes	-0.007	-0.193–0.180	(0.945)	-0.101	-0.299–0.097	(0.318)
>60 minutes	0.059	-0.152–0.270	(0.581)	-0.046	-0.478–0.387	(0.836)
Mid-Day Outdoor Time (0 minutes as reference)						
1–30 minutes	-0.147	-0.231 - -0.063	(0.001)	-0.091	-0.182 - -0.000	(0.049)
31–60 minutes	-0.354	-0.490 - -0.217	(< 0.001)	-0.254	-0.402 - -0.106	(0.001)
>60 minutes	-0.317	-0.484 - -0.149	(< 0.001)	-0.196	-0.377 - -0.014	(0.035)
After-School Outdoor Time (0 minutes as reference)						
1–30 minutes	0.041	-0.051–0.133	(0.381)	-0.002	-0.101–0.097	(0.965)
31–60 minutes	0.131	0.012–0.251	(0.030)	-0.004	-0.135–0.127	(0.949)
>60 minutes	0.131	-0.001–0.263	(0.053)	-0.013	-0.159–0.134	(0.866)

a Confidence intervals and p-values are based on robust standard errors adjusted for clustering at the school level.

Regarding other potential correlates of uncorrected vision and myopia, after controlling for school grade, there was no association between older age and UCVA. Girls had greater risk of worse vision and myopia than boys (Tables 2 and 3). Having both parents out-migrated for work significantly increased the risk of myopia in multivariate models (OR -0.155, P = .017) (Table 3), but was not significantly associated with UCVA (Table 2).

None of the parental education indicators were associated with UCVA or myopia risk in multivariate models, though either parent finishing high school was significantly associated with worse UCVA in the univariate analysis. Compared with living at home, living in a school dormitory was negatively correlated with UCVA and myopia, while other residence types were unassociated with vision or refractive error (Tables 2 and 3).

## Discussion

Our models of UCVA and myopia both point to consistent conclusions that time using computers and smartphones is associated with more myopic refractive error, while television viewing and after-school study are not. Evidence about the impact of smart phone and computer use on myopia has been inconsistent[13,14,23,24]. It may be that, as prevalence of use of these devices among school-age children continues to rise in China and elsewhere, the association is becoming clearer. It should be noted, though, that in the present cohort, daily use of both smartphones and computers was less common than the frequency of television watching and after-school study. Given the greater expected viewing distance for television, our failure to find an association with visual acuity or myopia, as opposed to computers and smartphones (generally used at closer distances), is consistent with the prevailing hypothesis that viewing distance plays a role in the influence of near work on myopia, due perhaps to greater peripheral defocus at closer working distance[25]. We speculate that the lack of an observed association between after-school study time and myopia or decline in vision might be due to afternoon study and tutorial classes, which some children may not have considered as “reading after school”. Once again, evidence on the impact of reading and studying on myopia incidence and progression has been somewhat inconsistent[13,14].

Recent trial evidence strongly suggests a causal association between increased time outdoors and decreased incidence of myopia[8,9]. However, the mechanism for the protective effect of outdoor time is still not definitively understood, with both increased exposure to bright light and decreased time focusing at near targets having been proposed. Our finding that statistically significant associations between outdoor time and reduced myopia were found only for the midday interval, and not before or after school, may support the hypothesis that light intensity plays a crucial role in the protective effects of outdoor time, corroborating results from animal experiments[26,27]. Time of day has not previously been identified as an important aspect when considering outdoor time as a mediator of myopia risk. Parts of China, including the current settings in our sample province, are at similar latitudes to areas with high sun exposure[28]; however, it may be that other factors such as weather and air pollution[29] partially block sunlight in China, requiring exposure at the brightest midday hours for protection against UCVA and leading in part to China’s high myopia prevalence[30,31].

Our findings suggest a potential anti-myopia intervention of encouraging more outdoor activities during the noon hours when illumination is greatest. Such strategies would be practical in China, due to the widespread practice of offering a noon break of up to 2 hours in Chinese schools, during which many children return home to rest. Strategies to exploit this noon break in order to increase sunlight exposure would have to contend with the cultural norm of sleeping during this time, and would also need to address issues such as protecting children’s skin from bright sunlight. Further evidence of the greater effectiveness of noon-time outdoor activity in protecting against myopia is needed in other settings.

Strengths of this study include its population-based nature and large size. The “diary” approach explored the effects of time spent on a variety of activities, including various types of near work and outdoor activities at different times of day. This alleviates concerns of substitution effects as confounding factors. There is also a clear distinction between noon-time outdoor activity and other outdoor activities in our data.

Some caution is necessary in interpreting our results. Self-reported recall data, as adopted by most studies of visual acuity[3], depend on the reliability of informants[32–34]; this issue may be greater when younger children are involved, as in the present case. Given the resource limitations to researchers following a large cohort of young children for long periods of time, the self-reported recall was determined to be the best method for our study’s visual acuity data collection. Parents' refraction data are often included to explain the incidence and progression of myopia[1], while resources to refract parents are rarely available in studies of children's refractive error, glasses wear can be used as surrogate for myopia in high resource settings, but this is less useful in rural China, where few who need glasses wear them. Though a large number of schools (over 250) were involved, all were selected from two adjacent areas in western China, and application of our findings to other settings must be made with caution.

Even with these limitations, our findings offer a novel evidence supporting the protective effects of increased outdoor time is protective against myopia. If further work confirms our assumption that higher levels of exposure to brighter lights during noontime is an effective method of myopia prevention, this may open the door to entirely-new myopia prevention strategies, such as the use of bright artificial lights in classrooms and various architectural accommodations to increase children’s exposure to higher levels of natural light.

## Supporting information

S1 File Related survey questions. (ZIP)
