# Supplementary material for: Impact of various types of near work and time spent outdoors at different times of day on visual acuity and refractive error among Chinese school-going children
Source: PLoS One. 2019 Apr 26;14(4):e0215827. doi: 10.1371/journal.pone.0215827 (PMC6485919; doi:10.1371/journal.pone.0215827)
Supplement: S1 File — (ZIP) [file pone.0215827.s001.zip › Related survey questions/Related survey questions-EN .docx]

**Enumerator number: _____________ Survey date:________________**

| **_______________District** | **_____________Street/Township** | |
| --- | --- | --- |
| **School** | **________________Year** | **_____________class** |
|  |  |  |

1. Basic Information

| **Questions** |  | **Answers** |
| --- | --- | --- |
| 1. What is your gender? | 1=Male 2=Female |  |
| 1. What is your age? | Year |  |
| 1. What type of your Hukou is? | 1= Rural Hukou  2=Urban Hukou  3= Don’t have Hukou |  |
| 1. Where did you live during last semester? | 1=Home  2= School dormitory  3= Relative’s home  4= Rental home  5= Other |  |
| 1. Did your father live at home during last semester? | 1=Yes； 2=No |  |
| 1. Did your mother live at home during last semester? | 1=Yes； 2=No |  |
| 1. What is your father’s education level? | 1=No schooling  2=Primary school  3=Junior high school  4=High school/professional high school  5=Professional college  6=Higher than college |  |
| 1. What is your mother’s education level? | 1=No schooling  2=Primary school  3=Junior high school  4=High school/professional high school  5=Professional college  6=Higher than college |  |

1. Time-using Status

| Questions |  | Answers |
| --- | --- | --- |
| 1. How much time do you spent on computer throughout the day? | 1=0 minutes；  2=Less than half hour (1 to 30 minutes)；  3=More than half hour and less than one hour (31 to 60 minutes)；  4 More than one hour (> 60 minutes) |  |
| 1. How much time do you spent on smartphone throughout the day? | 1=0 minutes；  2=Less than half hour (1 to 30 minutes)；  3=More than half hour and less than one hour (31 to 60 minutes)；  4 More than one hour (> 60 minutes) |  |
| 1. How much time do you spent on television viewing throughout the day? | 1=0 minutes；  2=Less than half hour (1 to 30 minutes)；  3=More than half hour and less than one hour (31 to 60 minutes)；  4 More than one hour (> 60 minutes) |  |
| 1. How much time do you spent on study or reading after school throughout the day? | 1=0 minutes；  2=Less than half hour (1 to 30 minutes)；  3=More than half hour and less than one hour (31 to 60 minutes)；  4 More than one hour (> 60 minutes) |  |
| 1. How much time do you spent outdoors before school throughout a day? (playing, running or farming work) | 1=0 minutes；  2=Less than half hour (1 to 30 minutes)；  3=More than half hour and less than one hour (31 to 60 minutes)；  4 More than one hour (> 60 minutes) |  |
| 1. How much time do you spent outdoors around midday throughout a day? (playing, running or farming work) | 1=0 minutes；  2=Less than half hour (1 to 30 minutes)；  3=More than half hour and less than one hour (31 to 60 minutes)；  4 More than one hour (> 60 minutes) |  |
| 1. How much time do you spent outdoors after school and before dark throughout a day? (playing, running or farming work) | 1=0 minutes；  2=Less than half hour (1 to 30 minutes)；  3=More than half hour and less than one hour (31 to 60 minutes)；  4 More than one hour (> 60 minutes) |  |

1. Family Assets Status

| Assets | 1=Yes；2=No | Assets | 1=Yes；2=No |
| --- | --- | --- | --- |
| 1. Automobile |  | 1. Camera |  |
| 1. Truck |  | 1. Washing Machine |  |
| 1. Motor Bike |  | 1. Air Conditioner |  |
| 1. Tractor |  | 1. Water Heater |  |
| 1. Farming Equipment |  | 1. Gas Stove |  |
| 1. Computer |  | 1. Kitchen Ventilator |  |
| 1. Internet |  | 1. Refrigerator |  |
| 1. Television |  | 1. Flushable Toilet |  |
