# Supplementary material for: Impact of various types of near work and time spent outdoors at different times of day on visual acuity and refractive error among Chinese school-going children
Source: PLoS One. 2019 Apr 26;14(4):e0215827. doi: 10.1371/journal.pone.0215827 (PMC6485919; doi:10.1371/journal.pone.0215827)
Supplement: S1 File — (ZIP) [file pone.0215827.s001.zip › Related survey questions/Related survey questions-CN.doc]

**_______________市_______________县________________乡/镇**

**________________小学____________年级____________班**

# 一、基本信息

| **问题** | **选项** | **答案** |
| --- | --- | --- |
| 1.你的性别？ | 1=男 2=女 |  |
| 2.你几岁了？ | 周岁 |  |
| 3.你的户口类型？ | 1=农村户口；  2=城镇户口；3=没有户口 |  |
| 4.上个学期你大部分时间住哪里？ | 1=家里 2=学校  3=学校附近亲戚家  4=在学校附近租房  5=其他，请说明。 |  |
| 5.上个学期，你爸爸大部分时间在家住吗？ | 1=是； 2=否 |  |
| 6.上个学期，你妈妈大部分时间在家住吗？ | 1=是； 2=否 |  |
| 7.你爸爸的文化程度？ | 1=没上过学 2=小学  3=初中 4=高中或中专  5=大专 6=大学及以上 |  |
| 8.你妈妈的文化程度？ | 1=没上过学 2=小学  3=初中 4=高中或中专  5=大专 6=大学及以上 |  |

# 二、用眼时间

| **问题** | **选项** | **答案** |
| --- | --- | --- |
| 9.你每天使用电脑的时间？ | 1=不用电脑； 2=半小时以内；  3=半小时到一小时； 4=一小时及以上 |  |
| 10.你每天玩手机的时间？ | 1=不玩手机； 2=半小时以内；  3=半小时到一小时； 4=一小时及以上 |  |
| 11.你每天看电视的时间？ | 1=不看电视； 2=半小时以内；  3=半小时到一小时； 4=一小时及以上 |  |
| 12.你每天放学后看书、写作业的时间？ | 1=不看书、写作业； 2=半小时以内；  3=半小时到一小时； 4=一小时及以上 |  |
| 13.你起床后，到校前，花多少时间在室外（屋子外）活动（如干农活、玩耍、晨跑等）？ | 1=不到室外活动； 2=半小时以内；  3=半小时到一小时； 4=一小时以上； |  |
| 14.中午休息时，你进行多长时间的室外活动（包括运动、玩耍、上街等）？ | 1=不到室外活动； 2=半小时以内；  3=半小时到一小时； 4=一小时以上； |  |
| 15.你在放学后，天黑前这段时间，花多少时间进行室外活动（包括运动、玩耍、上街等）？ | 1=不到室外活动； 2=半小时以内；  3=半小时到一小时； 4=一小时以上； |  |

# 七、家庭资产拥有情况

| 资产类别 | 1=有；2=没有 | 资产类别 | 1=有；2=没有 |
| --- | --- | --- | --- |
| 1. 小汽车 |  | 2. 照相机 |  |
| 3. 货车 |  | 4. 洗衣机 |  |
| 5. 摩托车或电动车 |  | 6. 空调 |  |
| 7. 拖拉机 |  | 8. 热水器 |  |
| 9. 大型农机具 |  | 10. 煤气/液化气炉具 |  |
| 11. 电脑 |  | 12. 抽油烟机 |  |
| 13. 拉网线 |  | 14. 电冰箱或冰柜 |  |
| 15. 电视机 |  | 16. 抽水马桶 |  |
